# Spectral and Spatial-Based Classification for Broad-Scale Land Cover Mapping Based on Logistic Regression

**DOI:** 10.3390/s8128067

**Published:** 2008-12-08

**Authors:** Georgios Mallinis, Nikos Koutsias

**Affiliations:** 1 Department of Forestry & Management of the Environment and Natural Resources, Democritus University of Thrace, GR-68200 Orestiada, Greece; E-Mail: gmallin@fmenr.duth.gr; 2 Department of Environmental and Natural Resources Management, University of Ioannina, Seferi 2, GR-30100 Agrinio, Greece

**Keywords:** Land cover mapping, logistic regression, autocovariate, texture

## Abstract

Improvement of satellite sensor characteristics motivates the development of new techniques for satellite image classification. Spatial information seems to be critical in classification processes, especially for heterogeneous and complex landscapes such as those observed in the Mediterranean basin. In our study, a spectral classification method of a LANDSAT-5 TM imagery that uses several binomial logistic regression models was developed, evaluated and compared to the familiar parametric maximum likelihood algorithm. The classification approach based on logistic regression modelling was extended to a contextual one by using autocovariates to consider spatial dependencies of every pixel with its neighbours. Finally, the maximum likelihood algorithm was upgraded to contextual by considering typicality, a measure which indicates the strength of class membership. The use of logistic regression for broad-scale land cover classification presented higher overall accuracy (75.61%), although not statistically significant, than the maximum likelihood algorithm (64.23%), even when the latter was refined following a spatial approach based on Mahalanobis distance (66.67%). However, the consideration of the spatial autocovariate in the logistic models significantly improved the fit of the models and increased the overall accuracy from 75.61% to 80.49%.

## 1. Introduction

A major part of research in satellite remote sensing is dedicated to the optimization of computer-aided classification processes for identifying and mapping various land cover/use types [1]. Land cover classification, which associates pixels or objects of remotely sensed data with specific land cover classes, is used for a plethora of applications including land resource planning, environmental change assessment, biodiversity conservation, and estimation of biophysical variables [2]. The classification results are organized into digital geo-databases and provided at multiple contents and scales. The recent improvements of satellite sensor characteristics (i.e. spatial, radiometric resolution) facilitate visual identification and recognition of various features and entities on the earth's surface. Apart from spectral information, experienced photo interpreters exploit spatial patterns and object arrangement based on the skills of the human brain to evaluate and recognize elements such as shape, size, pattern, shadow, colour tones, texture, association and site [3]. However, quantitative automatic digital classification techniques prevail over qualitative ones (i.e. photointerpretation), since the former utilize all the available range of spectral and spatial resolution that the human eye cannot easily recognize [4]. Ideal classification approaches cannot exist due to process complexity and a number of factors affecting classification outputs, such as the adopted classification scheme, spectral and spatial content of the imagery, the method of making class decision, and the classification unit. For example, different outputs could result from adopting different classification algorithms for the same training sets [4, 5]. Therefore, several efforts have been made in recent years to develop new classification techniques or adapt older ones [6-8].

Multispectral classification approaches that rely only on information extracted from single pixels (known as per-pixel spectral classifiers), allocate each pixel to an output classification class on the basis of a relative similarity (distance) of pixel's vector x to the mean vector of each class derived after user-selected training data. The range of spectral classifiers is extensive, including methods like the k-nearest neighbour [9], neural networks [10], regression models [11, 12], classification or decision trees [13, 14], and support vector machines [2]. Nevertheless, the Maximum Likelihood (ML) classifier is one of the most commonly used within the remote sensing community, and is also used as a standard for comparing other classifiers [15, 16]. The ML is a parametric classifier presupposing normal distributions, and is based on the variance-covariance matrix of the spectral responses of land cover classes for classifying a pixel. Unfortunately, insufficient, non-representative, or multimodal distributed training samples can introduce uncertainties resulting to misclassification when the ML is used. Another major drawback is the difficulty to integrate ML with other ancillary information, however this limitation is overcome by the use of modified prior probabilities [7].

Recently, progress achieved in the improvement of satellite sensor characteristics has lead to the existence of high resolution scene models (H-resolution) [17]. The increased ability of the spatial discriminator reveals the internal variability within targets causing occasional decrease in classification accuracy [18]. In the H-resolution scene model, the presence of spatial autocorrelation, which is the tendency of neighbouring pixels to present similar characteristics, is a potential problem. This problem promoted the development of new classification methods, known as contextual methods, which in addition to spectral information also consider the spatial information of the surrounding region of each pixel. The spatial information inherent in satellite data can be incorporated into the classification process either during the pre- and post-processing or prior to pixel labelling by using a contextual classifier [19]. The use of contextual classifiers usually results in a reduction of classification error rates [20-24]. However, contextual classification techniques involve a more complex decision process and tend to be more computationally intensive than spectral pattern recognition procedures [25].

In our study, a spectral land cover classification method of a LANDSAT-5 TM image that uses several binomial logistic regression models was developed, evaluated and compared to the familiar parametric Maximum Likelihood (ML) algorithm. The classification approach based on logistic regression modelling was extended to a contextual one by using autocovariates to consider spatial dependencies of every pixel with its neighbours. Autologistic regression modeling has been already introduced in remote sensing studies as a classification approach of satellite data, however it has been limited to binary classification schemes as for instance in flood zonation and burned land mapping [26, 27]. Finally, the ML classified image was upgraded to contextual by considering typicality, a measure which indicates the strength of class membership [28].

## 2. Study Area

**Figure 1.**
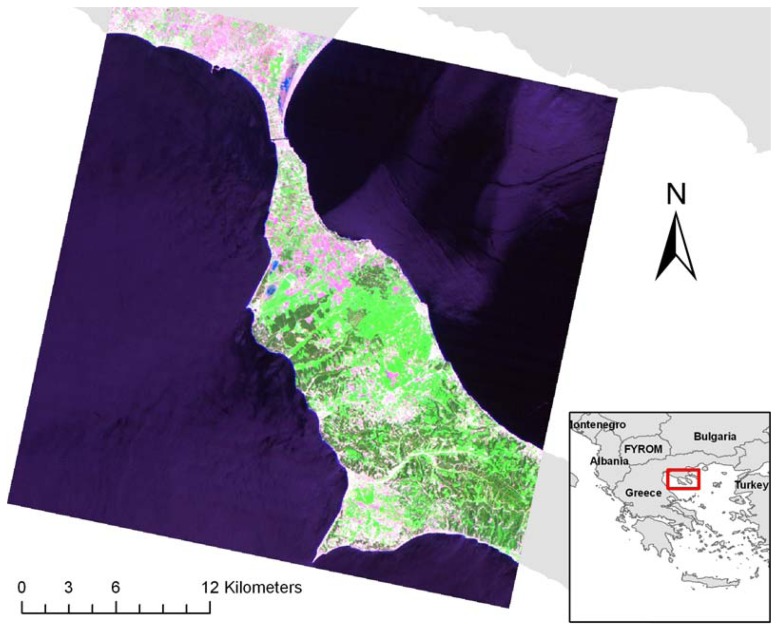
Location of the study area and colour composite (RGB: TM-743) of the Landsat TM image used in the study.

The study area is located in the uppermost part of the Kassandra Peninsula in NC Greece (Figure 1). Although each place is unique, depending on how environmental parameters and human actions are spatially integrated, our study area seems to be characteristic and representative of many landscape types found across Greece. The fragmented landscape consists of small patches of forested land, interchanged with agriculture and rangelands. The forested land mainly consists of Aleppo pine (*Pinus halepensis*) and dense shrubs (maquis), the latter which occasionally dominate the overstory within the stands. In these ecosystems, the main species present in the understory are *Quercus conferta*, *Quercus ilex and Pistacia lentiscus*. As in the majority of Mediterranean forests, the landscape is quite heterogeneous with regards to stand structure and composition.

The study area belongs to the Mediterranean type climate and the bioclimate is characterized as semi-arid with severe summer droughts and relatively high humidity throughout the year. The study area is subject to strong human influence and high tourist pressure which justifies the fragmented character of the landscape. The relief of the area is rather gentle with mild slopes resulting in non-severe topographic shading.

## 3. Materials and Methods

### 3.1. Image Data Preprocessing

A Landsat-5 Thematic Mapper image (path 184; row 032) was acquired on 11 May 1997. Haze removal was applied to the LANDSAT TM image by subtracting the amount by which each band's histogram is shifted from the origin due to atmospheric scattering. Following this dark pixel subtraction approach, the minimum value of each spectral channel was subtracted from each pixel brightness in that channel [29].

The Landsat TM image was orthorectified using 54 ground control points identified on 1:5,000 scale orthophotographs produced from a 1996-1997 national aerial photography campaign and a digital elevation model constructed from contour lines of 20 m increment. Orthorectification ensured that spatial inaccuracies caused by the irregular terrain would be minimized. The total root mean square error (RMS error) was about 0.6 of a pixel.

### 3.2. Data analysis

#### 3.2.1. Classification Scheme

Initially, six informational classes (Table 1) were identified after a field survey and photo interpretation of the orthophotographs. Training areas for each informational class were identified in the satellite imagery based on a field survey supported by GPS over characteristic locations in order to cover their spectral and spatial variability. In addition to simple descriptive statistics, the spectral consistency of the training areas was evaluated using a hierarchical cluster analysis procedure [30]. The Ward's method of clustering, which according to Milligan's [31] review of clustering techniques provides the best overall performance for most data sets, was selected along with the squared Euclidean distance as a measure of similarity between plots. In this clustering method, the distance between two clusters is the sum of squares between the two clusters summed over all the variables. At each stage of the clustering procedure, the within-cluster sum of squares is minimized over all separate or disjointed clusters [32].

#### 3.2.2. Maximum Likelihood (ML) Classification

The Maximum Likelihood classifier is based on the Gaussian estimate of the probability density function for each class. Assuming that the probabilities for all classes are equal, the probability density function is calculated for each class by [33]:

(1) $$p(z_{k}(x_{i})|c)=\frac{1}{{\left(2\pi \right)}^{k/2}{\left|V_{c}\right|}^{1/2}}\exp[-1/2M_{ci}]$$

where *p*(*z_k_*(*x_i_*)|*c*) is the probability density function for pixel *z_k_*(*x_i_*), as a member of class c at location *x_i_*, *k* is the vector of {*k* = 1,….*K*}wavebands, *V_c_* is the variance-covariance matrix for class *c*, and *M_ci_* is the Mahalanobis distance to class centroids:

(2) $$M_{ci}={\left(z_{k}(x_{i})-u_{kc}\right)}^{T}\;V_{c}(z_{k}(x_{i})-u_{kc})$$

where *u_kc_* is the vector of means in *K* wavebands of the particular class *c*, and *T* is a matrix transposition function.

Finally, each pixel is allocated to the class with the highest probability function or equivalent to the highest posteriori probability of membership obtained by the Bayes' Thorem under non-equal prior probabilities [34]:

(3) $$L(c|z_{k}(x_{i}))=\frac{P_{c}\cdot p(z_{k}(x_{i})|c)}{\overset{n}{\underset{r=1}{\text{∑}}}P_{r}\cdot p(z_{k}(x_{i})|r)}$$

where *L*(*c*|*z_k_*(*x_i_*)) is the conditional probability of pixel value *z_k_*(*x_i_*) belonging to class *c*, *P_c_* is the a priori probability of membership of class *c*, and there are *r* = *1*,…, *n* classes.

#### 3.2.3. Contextual Classification Based on Mahalanobis Distance

The classified image by ML algorithm was spatially weighted and reclassified using Mahalanobis distance [34]. Mahalanobis distance can be used to derive probability measures that indicate class membership, such as typicality probabilities, or it can be used straightforwardly [28, 35]. Typicality is better suited to identify misclassified pixels than the posteriori probability measure, because it represents a spectrally related measurement of classification confidence [36]. The pixels may have been classified to the class with the highest posteriori probability of membership, but they do not belong to that class. In the present study a 3 × 3 moving window with an inverse distance weighting was applied to the typicality values of the pixels within the imagery. The centre pixel was then assigned to the class presenting the highest sum of typicality values within the window.

#### 3.2.4. Logistic Regression Modelling

Multiple logistic regression modelling is used to predict a binary dichotomous variable Y from a set of independent explanatory variables by estimating the probability of the event's occurrence. The main assumption made in the logistic regression model is the linear relationship between the natural logarithm of the odds of the binary outcome (let Y take values 1 and 0) and the independent variables. In contrast to other multivariate statistical methods, no assumption of multivariate normality has to be satisfied.

Logistic regression may be proved useful for the classification of satellite remotely sensed data, especially when the independent variables (spectral observations) do not follow the normal distribution. The main consideration for implementing the logistic regression modelling into classification process is to express the classification problem in a binary dichotomous way, i.e. to consider the classification categories by two each time [11]. The equation for the logistic regression is expressed as:

(4) $$p_{i}=P(z_{k}(x_{i})|Y=1)=\frac{\exp(\beta_{o}+\overset{K}{\underset{k=1}{\text{∑}}}\beta_{k}z_{k}(x_{i}))}{1+\exp(\beta_{o}+\overset{K}{\underset{k=1}{\text{∑}}}\beta_{k}z_{k}(x_{i}))}$$

and

(5) $$\log it(P(z_{k}(x_{i})|Y=1))=\ln(\frac{p_{i}}{1-p_{i}})=\beta_{o}+\beta_{1}z_{1}(x_{i})+\beta_{2}z_{2}(x_{i})+\ldots .+\beta_{k}z_{k}(x_{i})$$

where the parameters *β_o_* and *β_k_* (*k* = 1,2,3.…*K*) of the *K* wavebands, are estimated using the ML method.

**Figure 2.**
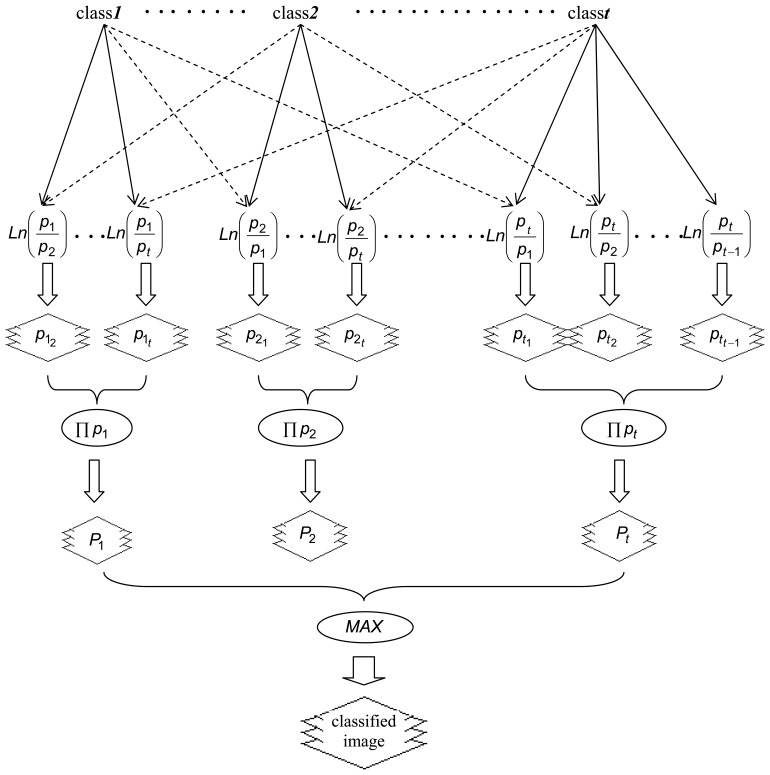
Flowchart of the classification approach based on logistic regression. Following the extraction of the training areas, *t-1* multiple logistic regression models were structured for each of the *t* classes. Using the estimated regression coefficients, binary images were produced and multiplied to derive an image of estimated probabilities for each class. Finally, pixels ware assigned to the class presenting the maximum probability.

The flowchart of classification using logistic regression modelling is presented in Figure 2 while the whole process is implemented by the following steps:

1. Assessment of training areas for each informational class and extraction of DN values. The spectral channels of TM imagery are perceived as independent variables while the land cover category is the dependent variable.
2. T groups (*t* classification classes) of *t-1* data files each, are formed and the main or baseline informational class is encoded to value 1. This set of files is the final input to the multiple logistic regression modelling process.
3. Using a forward multiple logistic procedure based on the likelihood ratio statistic, the coefficients of each model are estimated by considering three explanatory variables maximum. The independent variables of each model are the best-performing out of the seven available to discriminate each informational class.
4. The logistic regression models are applied and *t x (t-1)* new images are produced and organized in t groups according to the original file scheme. Within each group, the four images are combined through multiplication to produce a final probability image for each class.
5. The final classified image results by assigning to each pixel the land-cover category which corresponds to the highest probability value.

#### 3.2.5. Autologistic regression modeling

The autologistic regression model, which results after the addition of an autocovariate component to an ordinary logistic model, provides the opportunity to integrate spatial information into modelling. The integration of the autocovariate is based on the principle that adjacent pixels are more likely to belong to the same class. Therefore the probability of a candidate pixel to belong to a certain class, apart from considering the spectral information, also depends on whether the neighbouring pixels belong also to the specific class [26]. To implement autologistic modelling the autocovariate component has to be first estimated. Unfortunately, the autocovariate component cannot be estimated because the binary response variable is not initially available. To overcome this constraint, the autocovariate component can be estimated from the predicted probabilities of the binary response variable instead of the response variable itself [37].

The procedure includes the following steps [27, 37, 38]:

1. Estimation of the predicted probabilities of the binary response variable using the ordinary multiple logistic regression model.
2. Estimation of the autocovariate component from the predicted probabilities using a moving window. The autocovariate component is then incorporated into the ordinary multiple logistic regression model as a new covariate.
3. Estimation of the coefficients of the autologistic multiple regression model including the original covariates (three spectral channels) and the autocovariate component. The procedure can be repeated from step 2 using the estimated probabilities of step 3.

The formula of the autologistic regression model, based on the equation of the ordinary logistic regression for a grid of cells is the following:

(6) $$p_{i}=\frac{\exp(\beta_{o}+\overset{K}{\underset{k=1}{\text{∑}}}\beta_{k}z_{k}(x_{i})+b\overset{n,K}{\underset{g\neq i,k=1}{\text{∑}}}y_{kz}\theta_{k}z_{k}({\hat{x}}_{g})z_{k}(x_{i}))}{1+\exp(\beta_{o}+\overset{K}{\underset{k=1}{\text{∑}}}\beta_{k}z_{k}(x_{i})+b\overset{n,K}{\underset{g\neq i,k=1}{\text{∑}}}y_{kz}\theta_{k}z_{k}({\hat{x}}_{g})z_{k}(x_{i}))}$$

where *y_kz_* is the presence/absence value in the *z_k_*(*x_g_*) neighbouring cell for pixel *z_k_*(*x_i_*), n is the number of pixels of the contiguity matrix, *θ_k_z_k_*(*x_g_*)*z_k_*(*x_i_*) is the weighting distance function between the target pixel *z_k_*(*x_i_*) and pixel *z_k_*(*x_g_*), and b is the estimated autologistic regression coefficient for the autocovariate.

In our study development of the autologistic regression models was made by maintaining the same covariates (spectral bands) as the original logistic models, in order to test their relative significance and validity. Only eight nearest neighbours were considered and an inverse distance weighting was applied.

### 3.3. Assessment of the different classification procedures

A stratified random sampling procedure was adopted to select a total of 123 points that were used to estimate the accuracy of the classification results. The majority of the reference samples were located by field survey, while areas difficult to visit were located through photo-interpretation of the available orthophotographs. Overall and individual per class accuracy (users and producers) and the Kappa coefficient of agreement were estimated.

A pairwise test statistic Z was also applied on the Kappa coefficient of agreement to statistically compare the results of the four classification schemes [39]:

(7) $$Z=|K_{1}-K_{2}|/\sqrt{\mathrm{var}K_{1}-\mathrm{var}K_{2}}$$

Furthermore, classification performance of the maximum likelihood and logistic regression was assessed by considering the standardized probabilities based on which the pixels were assigned to a specific class [8]. To apply this approach a common measurement scale of the probabilities has to be adopted. Therefore, a standardization procedure was applied so the sum of the standardized probabilities *L*(*i*|*X*) for every location *x_i_* belonging to classes *r* = *1*, …, *n* classes, is equal (i.e. 1 or 100). In the case of ML classification, standardized probabilities are identical to the posterior probabilities [40], while for logistic regression, the standardized probabilities can be calculated by the following formula:

(8) $$L(z_{k}(x_{i})|Y=1)=\frac{P_{i}}{\overset{r}{\underset{i=1}{\text{∑}}}P_{i}}$$

where *L*(*z_k_*(*x_i_*)|*Y*= 1)|*X*) is the standardized probability for the class *i*, and *P_i_* is the predicted value of occurrence for class *c* out of *r* = *1*,*2*,*3*…*t* classes at location *X_i_* (as calculated in paragraph 3.2.4.).

Finally, two landscape pattern metrics, the Mean Patch Size (MPS), a common fragmentation index [41], and Edge Density (ED), a robust metric [42], were estimated to quantify the spatial structure of the polygons resulting after conversion of the raster format classified images to vector format.

## 4. Results and Discussion

### 4.1. Purification of classification categories

**Figure 3.**
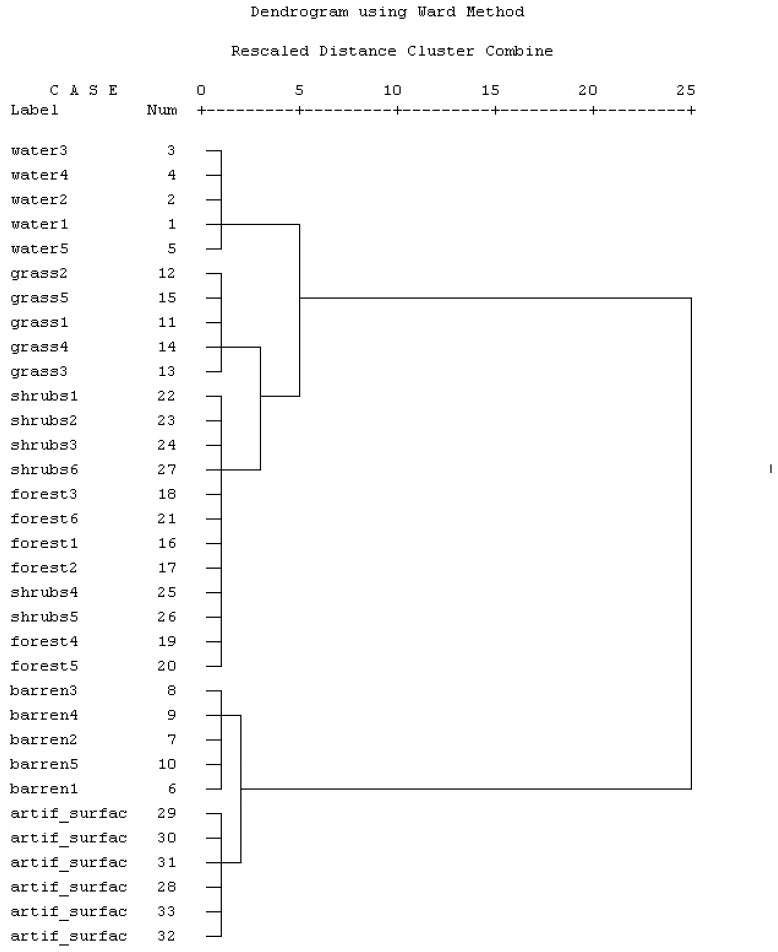
Hierarchical cluster analyses of the training areas originally delineated in the Landsat TM image. Shrublands and forests are clustered together in a very short distance, indicating spectral similarities which may create spectral confusion and misclassification.

Hierarchical cluster analysis results are presented in Figure 3 in dendrogram form, where the high similarity between the spectral signatures of forests and shrublands is very clear. The training plots of the two categories are clustered together in the same distance creating uncertainties for their successful discrimination and mapping. In our study area the forested land presents a very heterogeneous composition and structure similar to the majority of Mediterranean forest ecosystems.

**Figure 4.**
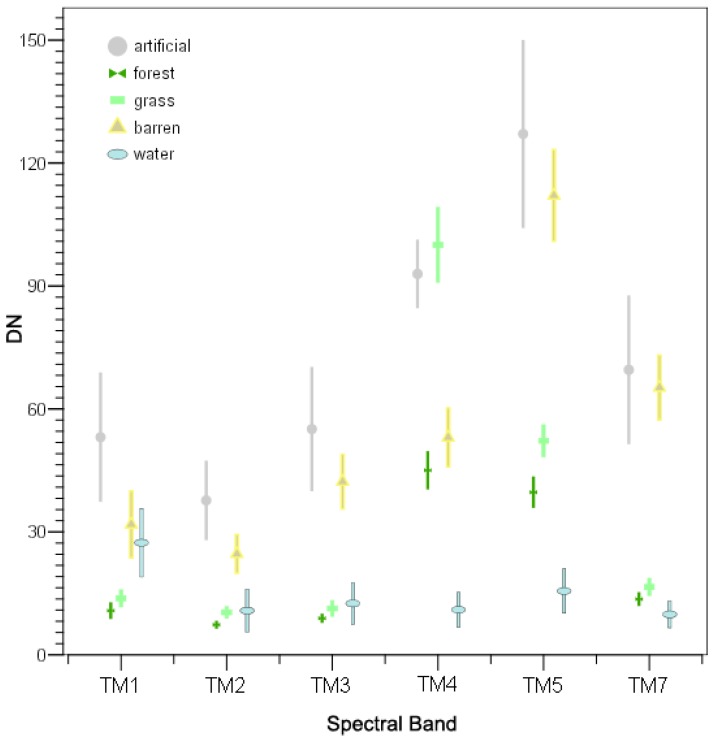
Descriptive statistics of the final classification scheme. Vertical bars extent 1 standard deviation around the mean.

When the stands are sparse and the foliage coverage is not dense, the reflectance of the broadleaved species of the understory contributes significantly to the total reflectance of the pixel and results in spectral responses similar to areas dominated by shrubs in the overstory [12]. Therefore, these two categories were grouped together into one single category-forest. A very good correspondence between the remaining categories and the suggested cluster analysis grouping can be seen. The mean and standard deviation values of the spectral classes after the merging are presented in Figure 4.

The overall accuracy of the image classified by the ML algorithm was 64.23%, while the accuracy of the image classified by multiple logistic regression was 75.61% (Table 2). Additionally, the achieved user's and producer's accuracy were in most cases higher in the latter classification approach. The Kappa coefficient of agreement for the classified image by the logistic regression method was significantly higher (0.68) than ML algorithm (0.56).

As observed in Table 3, the vast majority of the pixels (90%) were classified with high probabilities in both methods (over 90%) which implies a high degree of certainty for the classification results. However, in logistic regression a smaller fraction of pixels was classified with low probabilities (less than 0.5), which denotes that the classification of low discriminated categories is implemented at a higher confidence level.

**Figure 5.**
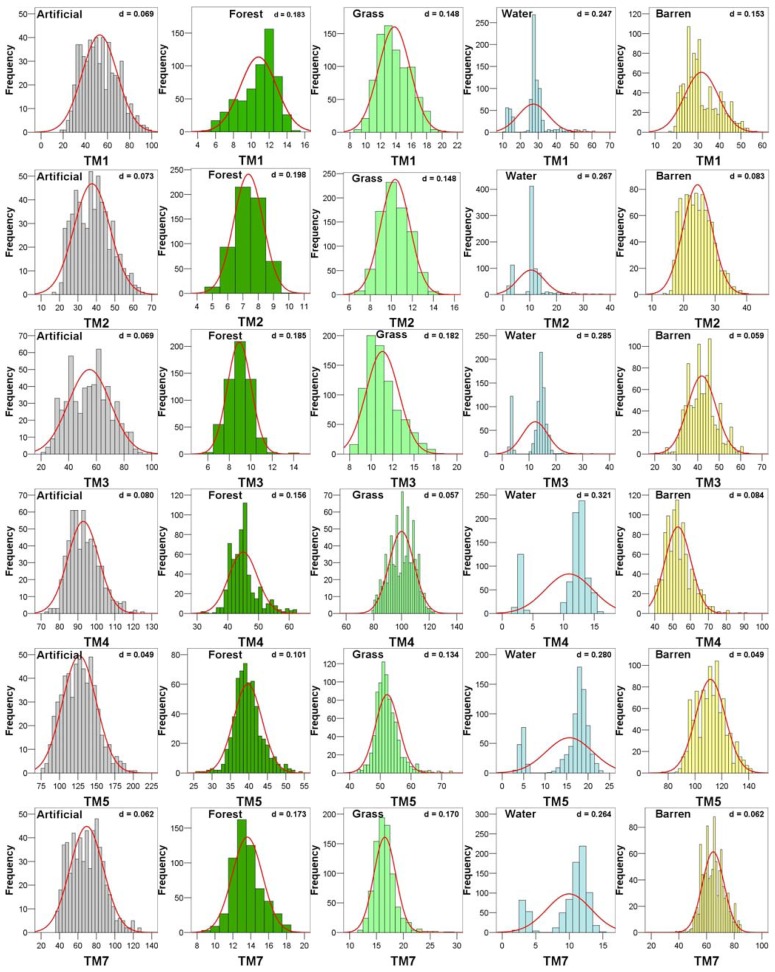
Observed frequency distributions and Kolmogorov-Smirnov values d calculated for each class. None of them was found to be normally distributed at 95 % confidence level according to the estimated d value.

Individual class accuracies were low for the classes “artificial surfaces” and “barren”, especially in the maximum likelihood based approaches. As it can be observed in Figure 4, these classes not only present spectral similarities between them but also they are less uniform since they present greater variability in their radiometric values. In addition, ML is a parametric classifier which presupposes normal distributions, while it is based on the variance-covariance matrix of the spectral responses of land cover classes for classifying a pixel. Unfortunately, multi-modal (e.g. water) or non-normally distributed data (e.g. artificial, barren) as observed in the broad-scale land cover types of our study (Figure 5) can introduce uncertainties which result further to misclassification. Previous studies have also shown the dependence of the classification performance to the composition and distribution of the classes [43]. On the other hand, logistic regression modeling is a non-parametric classification approach, which presupposes fewer statistical assumptions for its implementation compared to ML. Eventually, these can be underlying factors for explaining the low accuracies of the classification results of these classes.

The use of the typicality measure as a contextual refinement to ML algorithm improved the overall classification accuracy (66.67%) and the Kappa coefficient of agreement (0.59). The contextual process, which creates and uses information from the pre-defined neighboring pixels, estimates new probabilities for each pixel. While pixels may have been classified to a certain class with the highest posteriori probability of membership, the contextual approach improves classification results by assigning new classes to pixels that do not belong to this class using spatial information. Therefore, uncertainties occur in the original classification are expected to be reduced.

**Figure 6.**
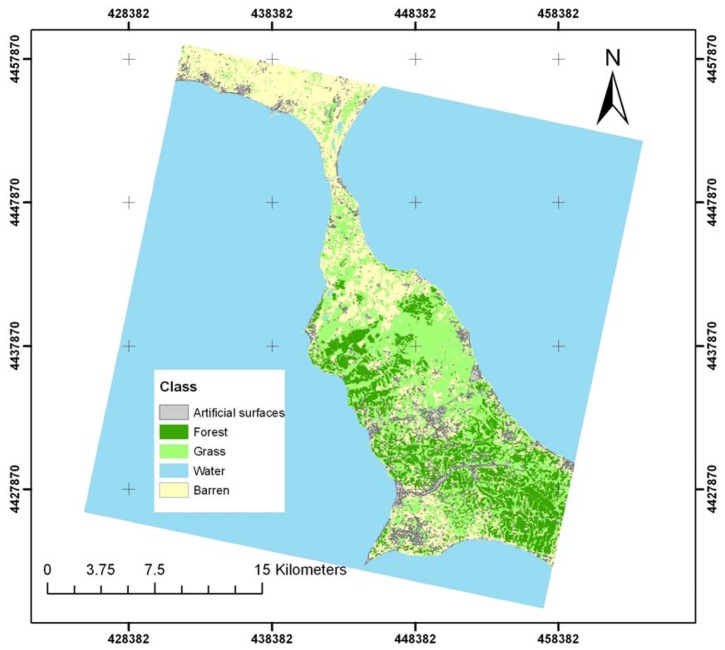
Land cover map using the autologistic regression modeling approach. Grid numbers in meters are in Greek grid projection.

Similarly, the consideration of the spatial autocovariate in the logistic models (Figure 6) significantly improved the fit of the models and increased the overall accuracy from 75.61% to 80.49% (Table 2). In the autologistic model, the probability of a candidate pixel belonging to a class depends on whether the neighbouring pixels belong also to that class. The autologistic approach follows the same principles utilized when applying a simple post-classification majority filter. However, the processing rule on which each method is based differs between the two. In the majority filter, only the number of pixels having the same value is considered, while in the autologistic model both the radiometric values of the pixels and the autocovariate component are taken into account [26].

In addition, a pairwise test statistic was applied to statistically compare the results of the four classification schemes based on the Kappa coefficient of agreement (Table 4). The value of 1.57, which results after the comparison of the ML classification with the logistic regression classification, does not exceed the crucial threshold of 1.96 (z-statistic at 95% confidence level) implying that the two methods are statistically non-significant. Instead, the comparison of the autologistic with the ML algorithm shows statistical differences, while the same finding stands after the comparison with the Mahalanobis-based post-classified image.

**Figure 7.**
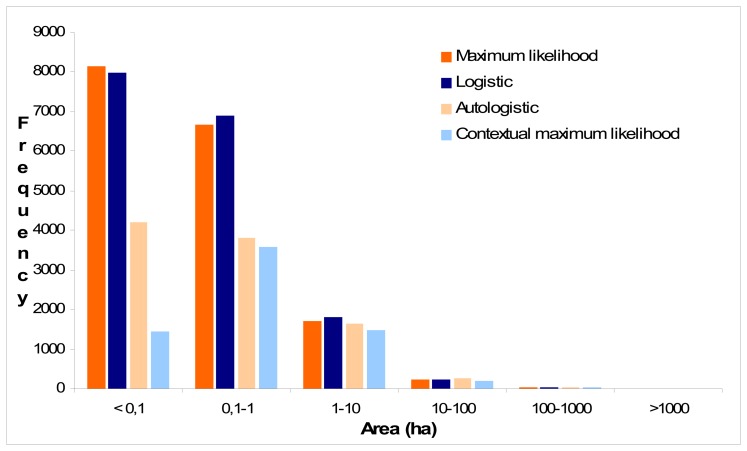
Frequency distribution of polygon size resulting after vectorization of the classified images.

The improvement of classification accuracy after incorporating the autocovariate in the original logistic classification approach and the post classification in maximum likelihood is justified by the lower number of polygons which results after the vectorization of the classified images (Figure 7). This is especially obvious for polygons smaller than 0.1 ha which approximates the area represented by one Landsat pixel. The reduction of the number of isolated pixels implies a reduction of the “salt and pepper” effect which is critical when the classification results are integrated in geographic databases or presented as cartographic outputs. One point shall be given emphasis is the arbitrary choice of the neighbourhood size and the weighting function prior to autocovariate estimation. An improper choice may lead to a substantial extent of generalization. However, it seems that in fragmented landscapes a small sized window might be more appropriate, particularly for medium sized pixels while larger windows are more appropriate at finer resolution satellite images [16].

**Figure 8.**
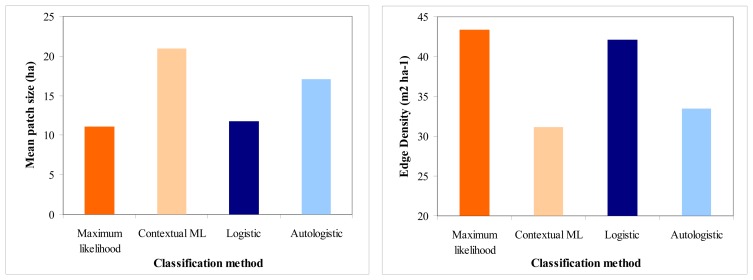
Landscape pattern metrics of the classified images of the four classification approaches.

Finally, landscape metrics estimated for the classification results (Figure 8) verified that the landscape patterns of the classified images were less fragmented when the spatial information was incorporated. Both contextual approaches resulted in less fragmented land cover maps, characterized by a larger mean patch size and smaller edge density values compared to those resulting from the maximum likelihood and logistic regression approaches.

## 5. Conclusions

The use of multiple logistic regression for broad-scale land cover classification proved to be more efficient than the well-known classification algorithm of maximum likelihood even when the latter was refined following a spatial approach based on Mahalanobis distance. The accuracy achieved by the multiple logistic regression approach (75.61%) confirms the possible use of this statistical technique in the classification of broad-scale land cover types using remotely sensed data. Parametric classifiers, such as ML, which presupposes normal distributions, can be insufficient and limited especially in cases where data present multimodal and not normal distributions. In such cases those traditional methods can be substituted by other statistical methods that do not require those assumptions, such as logistic regression. The laboursome and time demanding method of logistic regression can be overcome by the integration of a built-in routine to commercial image processing software.

The extension of the logistic approach to an autologistic one was very successful since it reduced the number of polygons resulted from the classified image and improved the overall accuracy (80.49%). Several classification methods which utilize only the spectral information of satellite sensor imagery are in certain cases insufficient due to spectral similarities of classes. In such cases, spatial information may be useful when considered to increase the limited spectral separability.

## Figures and Tables

**Table 1.** Land cover types in the study area and corresponding training areas.

**Land cover type**	**Description**	**Number of training plots / pixels**
Artificial surfaces	Urban areas and man-made structures (roads, camps)	6 / 572
Forest	Coniferous forests (*Pinus halepensis*)	6 / 632
Shrubs	Shrublands mixed with interspersed *P. halepensis* (maquis, including *Q. coccifera*, *Q. ilex* and *Arbutus unedo*)	5 / 575
Grass	Cultivated crops and pastures which at the time of image acquisition, due to the vegetation phenology and the area's climatic conditions, are in full bloom	6 / 812
Barren	Bare rocks, very sparsely vegetated areas, and non-cultivated farmlands	5 / 962
Water	Wetlands and sea	5 / 732

**Table 2.** Accuracy measures of the four classification methods, reference points and area extent of each land cover type of the final map resulted from the autologistic regression modeling approach.

		**1. Maximum likelihood**		**2. Contextual ML**		**3. Logistic regression**		**4. Autologistic regression**	
		
	Area of the map (km^2^)/Reference points	Producers	Users	Producers	Users	Producers	Users	Producers	Users
Artificial surfaces	37.9/13	100.00	27.08	100.00	28.26	38.46	55.56	46.15	40.00
Forest	48/29	82.76	88.89	89.66	89.66	89.66	86.67	86.21	96.15
Grass	93.3/32	62.50	83.33	68.75	88.00	75.00	75.00	87.50	82.35
Water	817.7/16	93.75	100.0	93.75	100.0	100.00	100.0	100.00	100.0
Barren	97/33	21.21	77.78	18.18	75.00	66.67	61.11	72.73	75.00
	Overall accuracy	64.23		66.67		75.61		80.49	
	**Kappa**	0.56		0.59		0.68		0.75	

**Table 3.** Posterior probabilities of the classified images estimated by the logistic regression and the maximum likelihood algorithm.

	**Maximum likelihood**			**Logistic regression**		
Probabilities threshold	Number of pixels	Percent (%)	Cumulative percent (%)	Number of pixels	Percent (%)	Cumulative percent (%)
0,1	32556	2.68	2.68	1514	0.12	0.12
0,2	35805	0.27	2.95	1514	0	0.12
0,3	39052	0.27	3.21	1514	0	0.12
0,4	42838	0.31	3.52	1716	0.02	0.14
0,5	47126	0.35	3.88	3780	0.17	0.31
0,6	52670	0.46	4.33	20393	1.37	1.68
0,7	59719	0.58	4.91	39632	1.58	3.26
0,8	69963	0.84	5.76	62503	1.88	5.14
0,9	90133	1.66	7.41	98830	2.99	8.13
1,0	1215609	92.59	100.00	1215609	91.87	100.00

**Table 4.** Significance matrix of the four classification approaches. Shaded cells indicate statistical significant differences at 95% confidence level.

	Maximum Likelihood (ML)	Logistic	Autologistic
**Logistic**	1.58		
**Autologistic**	2.55	0.93	
**Contextual ML**	0.40	1.28	2.30
